# Gambling: Exploring the Role of Gambling Motives, Attachment and Addictive Behaviours Among Adolescents and Young Women

**DOI:** 10.1007/s10899-022-10124-8

**Published:** 2022-05-17

**Authors:** L. Macía, A. Estévez, P. Jáuregui

**Affiliations:** Faculty of Health Sciences, Psychology Department, University of Deusto, Apartado 1, 48080 Bilbao, Spain

**Keywords:** Gambling, Addictive behaviours, Gambling motives, Attachment, Women, Adolescence

## Abstract

There is a growing body of research that seeks to understand the aetiology, consequences and risk factors associated with addictive behaviours in youths. However, research examining the specific profile of adolescent females is very limited. Therefore, the objectives of the present study were, firstly, to explore the differences between attachment, gambling motives (social enhancement and coping), positive and negative affect, and addictive behaviours (gambling, drugs, spending, alcohol and video games) in female adolescents with and without risk of gambling problems. Secondly, the relationships between attachment, gambling motives, positive and negative affect and addictive behaviours were analysed in the subsample of female adolescents with problem gambling Thirdly, we examine the predictive role of positive and negative affect, gambling motives, and attachment in the aforementioned addictive behaviours. The sample was composed of 351 adolescents and young women, of which 312 had no risk of gambling and 39 had gambling problems. The results obtained revealed higher scores in drugs, spending, maternal attachment, and all gambling motives in the group of gambling problems. Likewise, analyses showed that the relevance of the predictor variables (attachment, gambling motives, and affect) varied according to the addiction that was taken as a reference point (i.e., gambling, drugs, spending, alcohol and video games).Consequently, the identification of the possible vulnerability factors for each addiction could be useful in the design of prevention and treatment approaches. In addition, the need for integrated and holistic health- and social- care programmes are suggested in terms of sex and age.

## Introduction

Gambling Disorder (GD) is characterised by persistent and recurrent problematic gambling behaviour that leads to clinically significant damage (e.g. loss of job, deterioration of important personal relationships, indebtedness, anhedonia, emotional disorders, loss of control, sleep disorders, etc.) (American Psychiatric Association [APA], 2013). Previous studies in the area have shown that an early onset of gambling is associated with a greater predisposition towards the development of a GD in later life, as well as with a greater severity both of the problem itself and its subsequent negative consequences (Derevensky et al., 2003; Jiménez-Murcia et al., 2010; Sharman et al., 2019). A systematic review conducted by Calado et al., (2017a, b) indicates that problem gambling in the adolescent population ranges from 0.2 to 12.3%. However, research addressing this issue, on one hand, is not based on a homogeneous tool to measure gambling severity and, on the other hand, is based on samples of gamblers, not on the general population (e.g., from non-participation to gambling disorder). In addition, most of the research has focused on exploring the profile of the male gambler (Stark et al., 2012). Consequently, studies have yielded contradictory and limited results (Dowling, Merkouris, et al., 2017; Dowling, Shandley, et al., 2017). In fact, research is increasingly showing that the pattern of gambling may differ according to gender (Abbott et al., 2018; Sancho et al., 2019).

Gambling motives are a capital aetiological factor of GD, explaining an individual’s vulnerability to the development of addictive behaviour (Huic et al., 2017; Jáuregui et al., 2020). According to the model proposed by Stewart and Zack (2008), there are three main reasons for gambling: (a) enhancement motives (ENH), (b) coping motives (COP), and (c) social motives (SOC). Several studies have shown that adult women are more likely to use gambling as a coping mechanism to deal with worries and negative emotions (Lelonek-Kuleta, 2021). In the adult population, COP and ENH motives appear to be the main predictors of severe problem-gambling behaviour, whereas social motives seem to be related to non-pathological gambling (Barrault et al., 2019; Lambe et al., 2015).

In contrast to sex differences in gambling motivation in adults, studies on adolescents seem to show different patterns. In this vein, young people at risk or with problem gambling score higher on all gambling motives, including social motives, than those who do not have a gambling problem (Grande-Gosende et al., 2019). In fact, excitement, fun or socialisation are some of the main reasons why adolescents engage in gambling (Jáuregui & Estévez, 2019; Jáuregui et al., 2020; Neighbors et al., 2002). Currently, we have not found enough studies investigating sex differences in gambling motivation in adolescence. Nevertheless, impulsivity, especially affective impulsivity (i.e., the tendency to act rashly under the influence of intense positive and/or negative affective states), seems to be a transdiagnostic factor for addictions in this age group (Del Prete et al., 2017; Navas et al., 2017). In this line, studies suggest that the younger one is, the higher is one’s level of impulsivity, the greater the tendency towards immediate rewards and higher the difficulty in foreseeing the consequences of risky behaviour; which has been associated with a neurobiological susceptibility that is characteristic of youth, who have certain brain areas that are still developing (Estévez et al., 2015; Lockwood et al., 2017).

On the other hand, adolescent males and females with severe gambling problems show a remarkably similar prevalence of comorbid mental health problems, highlighting mood and anxiety disorders, substance use disorders, and the frequency and severity of gambling, compared to adolescents without such problems (Ellenbogen et al., 2007; Estévez et al., 2020; Estévez et al., 2020; Estévez et al., 2020; Estévez, Jáuregui, et al., 2020; Jáuregui et al., 2020). Regarding the profile of adolescent females who suffer from gambling addiction, previous studies indicate that, compared to the general female population, they have significantly higher rates of gambling, anxious-depressive symptoms, eating disorders, compulsive buying, and alcohol and drug use (Afifi et al., 2010; Boughton & Falenchuk, 2007). Moreover, although women tend to initiate addictive behaviours later than do men, the progression towards the development of dependence is quicker for them (Fonseca et al., 2021). This phenomenon, known as "telescoping", seems to occur both in substance- and non-substance-related addictions (e.g., alcohol, cannabis, cocaine, opioids, gambling, etc.), and it could explain why women enter treatment with more severe behavioural, family, psychological, and social problems (Slutske et al., 2015). However, differences in rates of addictive behaviours have been more closely related to gender/cultural environment than to biological factors (Fonseca et al., 2021). Therefore, as addictive behaviours become more feminised and normalised among the female population, we observe an increasing prevalence and an earlier age of onset of those behaviours, highlighting GD, video game addiction, or tobacco and alcohol abuse, among others (Ait-Daoud et al., 2017; Erol & Karpyak, 2015; Estévez et al., 2020b, 2020c, 2020d; Estévez, Jáuregui, et al., 2020; Lopez-Fernandez et al., 2019).

Among the risk and protective factors for addiction in adolescence, the relationship with the family and peers is particularly important (Estévez et al., 2019). In this sense, authors such as Estévez et al. (2017) propose that addictive behaviours in adolescence could be linked to a need for relational satisfaction. In fact, dependency behaviours have been considered by some authors as an attachment disorder, finding negative relationships between secure attachment style and acting-out behaviours in adolescents (Calado et al., 2017a, 2017b; Schimmenti et al., 2012). Teng et al. (2020) report that attachment to parents and peers is negatively associated with involvement in addictive problem behaviours (Estévez et al., 2020; Estévez et al., 2020; Estévez et al., 2020; Estévez, Jáuregui, et al., 2020; Monacis et al., 2017). In fact, an insecure attachment style has been found to be a predictor of Internet abuse, Internet gambling disorder, GD, or alcohol and drug abuse disorders in young people (Estévez et al., 2017; Schindler, 2019; Teng et al., 2020). However, the involvement of paternal and maternal attachment according to the addictive behaviour and gender has been unexplored in this age group.

In this sense, attachment constitutes a fundamental factor in determining the abilities, resources and skills an individual will develop in order to cope with everyday life (Mikulincer, & Shaver, 2007). However, not all attachment interactions are resolved in a functional way. Sometimes the attachment figure is emotionally unavailable or the infant may judge the attachment figure to be unavailable for her needs. Due to the failure to seek security in the caregiving figure, the infant's regulatory system may develop secondary regulatory strategies. In addictions, it has been suggested the self-medication theory which formulates addictive behaviours as a way to alleviate and regulate mood states, even as a way of emotional avoidance (Khantzian, 1985). There is evidence pointing out the expectation among people with GD of alleviating negative mood states and generating positive ones through gambling.

### Objectives

In summary, while there is a growing body of research examining the aetiology, prevalence, and risk factors associated with adolescent gambling disorder, research examining sex differences, that is, the profile of young women and adolescents with problem gambling, is very limited. Therefore, the aim of this study is, firstly, to explore the differences between groups of problem and non-problem gamblers in attachment, gambling motives (social, enhancement and coping), positive and negative affect, and addictive behaviours (gambling, drugs, spending, alcohol and video games). Secondly, we wish to analyse the relationship between these variables in the subsample of at-risk and possible problem gamblers. Thirdly, we intend to examine the predictive role of positive and negative affect, gambling motives, and attachment in the aforementioned addictive behaviours.

### Hypothesis

Firstly, young women and adolescents with gambling problems are expected to have higher scores on gambling, gambling motives (ENH, COP, and SOC), positive and negative affectivity, attachment difficulties and other addictive behaviours than those without gambling problems. Secondly, gambling behaviour is expected to be positively related to other comorbid addictive behaviours, gambling motives (ENH, COP, and SOC), and positive and negative affect; whereas a negative relationship is expected between addictive behaviours and attachment relationships. Finally, the role of the predictor variables (affectivity, gambling motives and attachment) is also expected to vary according to addiction type (gambling, video games, spending, drugs and alcohol) in the subsample of adolescents and young women with risk and problem gambling.

## Methods

### Participants

A convenience sample was recruited. The study sample included 351 young women and adolescents aged between 12 and 26 years, recruited from educational centers (that is, secondary schools and professional training centers), and treatment centers for pathological gambling associated with FEJAR (Spanish Federation of Rehabilitated Gamblers). All the educational centers from Basque Country (Spain) that comprised participants from the age range of the study were contacted, as well as all the treatment centers for pathological gambling associated with FEJAR (Spain). Finally, 10 educational centers from Basque Country as well as from other autonomous communities from Spain that contacted the research team and showed interest in the study, as well as participants recruited from FEJAR centers, participated in the study.

In this study, participants were divided into two subsamples based on their scores on the South Oaks Gambling Screen Revised for Adolescents (SOGS-RA; Winters et al., 1993): (a) the group of problem gamblers included women at risk or presenting possible problem gambling from both the association centres and the general sample, i.e. subclinical sample (scored of 2 or more), and (b) the group of women without gambling problems from the general sample (scored between 0 and 2, including non-gamblers).

**Table:** 

	Gambling problems group	Group without gambling problems
	N = 39	N = 312
Mean age	16.83 (*SD* = 3.85)	15.36 (*SD* = 1.86)
*Educational level*		
No studies	2.6%	0.6%
Primary studies	23.1%	35.3%
Secondary studies	46.2%	46.2%
High school	7.7%	8.7%
Technical and vocational training	15.4%	8%
University studies	5.1%	1.3%
*Employment*		
Full-time workers	15.4%	0.6%
Students	82.1%	98.1%
Unemployed	2.6%	0.3%
Studied and worked at the same time	–	1%

### Measures

#### Gambling Disorder

South Oaks Gambling Screen-Revised for Adolescents (SOGS-RA; Winters et al., 1993). Adapted to Spanish by Secades and Villa (1998). This instrument is composed of 12 items describing gambling behaviour over the last twelve months. All items have a dichotomous response option (i.e., "*yes*" or "*no*"), with the exception of Item 1, which has four response options (e.g. “*Has your betting money ever caused any problems for you such as arguments with family and friends, or problems at school or work?* or *Have you ever gambled more than you had planned to?”*)*.* The SOGS-RA is interpreted as follows: 0–1, no problem gambling; 2–3, risk of problem gambling; 4 or more, possible gambling disorder. The criteria used by the SOGS-RA for the detection of gambling problems are similar to the SOGS designed for adults (Lesieur & Blume, 1987) but the risk category combines current symptoms with those indicating the development of a later gambling problem. The original instrument has adequate psychometric properties (Cronbach's alpha = 0.81). In the present study, Cronbach's alpha was 0.91.

#### Drugs, Alcohol, Video Games, and Spending

MULTICAGE CAD-4 (Pedrero-Pérez et al., 2007). This instrument is designed to detect addictive behaviours, both substance and behavioural, as well as the social problems associated with them. The scale is composed of 32 items, which are grouped into eight types of addictive behaviours: (1) alcohol use disorder (2) drug use disorder; (3) gambling disorder; (4) video game addiction; (5) Internet abuse; (6) compulsive spending; (7) sex addiction; (8) eating disorders. In this study, alcohol, drugs, video games and spending were assessed. The items have a dichotomous response option (i.e., "yes" or "no"). Each addictive behaviour is scored on the basis of four questions replicating the CAGE questionnaire (Hayfield et al., 1974), including self-perception of the problem, perception of close family and peer relationships, feelings of guilt and withdrawal, and impulse control-symptoms. Interpretation of the scores is as follows: 0–1, no addiction problem; 2, risk of addiction; 3, likely to present addiction; 4, addiction problem. Internal consistency was satisfactory (Cronbach's α = 0.86 for the overall instrument, and above 0.70 for each subscale). The instrument detects between 90 and 100% of already diagnosed cases. In this study, Cronbach's alpha ranged between 0.60 and 0.90.

#### Attachment

The Inventory of Parent and Peer Attachment (IPPA; Armsden & Greenberg, 1987). Adapted to Spanish by Gallarín and Alonso-Arbiol (2013). This is a three-part self-report questionnaire that assesses adolescents' attachment to mother, father and peers. Attachment to each specific figure (e.g., mother) is assessed through a 16-item subscale with a 5-point Likert-type response (1 = *never*; 5 = *always*). The aggregate score for each person represents the overall strength of attachment, where high scores indicate high-quality attachment, and low scores indicate insecure attachment bonds. The original instrument has adequate psychometric properties for each specific figure as well as for the final score. The Spanish version shows optimal levels of Cronbach's alpha coefficients for mother (α = 0.87), father (α = 0.88) and peers (α = 0.93). In the present study, Cronbach's alpha coefficients ranged between 0.92 and 0.97.

#### Gambling Motives

Gambling Motives Questionnaire (GMQ; Stewart & Zack, 2008). Spanish adaptation by Jáuregui et al. (2018). The questionnaire assesses 15 reasons why people gamble, divided into three subscales of five items each: (1) Enhancement motives (ENH): refers to internal positive reinforcement to increase positive emotions (e.g., *To get an "intense" feeling*); (2) Coping motives (COP): alludes to internal negative reinforcement, aiming to avoid or ameliorate negative emotions (e.g., "*Because it helps you when you feel nervous or depressed*"); (3) Social motives (SOC): refers to external positive reinforcement, mainly social affiliation (e.g., "*Because it's what most of your friends do when they get together*"). Each item is an adaptation of the Drinking Motive Questionnaire (Cooper et al., 1992). The GMQ items have a 4-point Likert-type response ranging from 1 (*never/almost never*) to 4 (*almost always*). All subscales showed good internal consistency (α > 0.80). In the present study, Cronbach's alpha ranged from 0.82 to 0.90.

#### Affect

Positive and Negative Affect Schedule (PANAS; Watson et al., 1988), adapted to Spanish by Sandín et al. (1999). The scale is composed of 20 words describing different emotions and feelings, divided into two main subscales with 10 items each: Positive Affect (PA) and Negative Affect (NA). The respondent is asked to indicate whether they experienced any of the described emotions/affections now or in the last two weeks on a five-point Likert scale (1 = *not at all/very slightly* to 5 = *very much*). The total score for each subscale is the sum of the 10 items that make up the subscale, so the scores for each subscale range from 10 to 50 points. Higher scores indicate a greater presence of the specific affect. Both subscales, positive and negative affect, have good psychometric indices (α = 0.85 and 0.89, respectively). In this study, internal consistency was high now and at two weeks (α = 0.87).

### Procedure

Both paper-and-pencil and online questionnaires were administered. The vast majority of participants made paper-and-pencil assessments, whereas a minority completed the survey through a dedicated online link under the supervision of their teacher. The questionnaire included general information about the main goals of the study. It was also made clear that there were no right or wrong responses and that participants could email the research team if they wanted further information about the study. To be eligible, participants were requested to give informed consent. Parental consent was sought for those younger than 18 years of age.

Confidentiality, anonymity, and voluntary participation were ensured for all participants. Researchers’ contact information was provided for those who required it. The participants did not receive any compensation for participating. The Institutional Review Board approved the study (ETK-26/17-18). The schools participating in the study have received feedback about the results of the research.

### Data Analyses

Firstly, the mean differences between the group of problem gamblers and non-gamblers were analysed using Student's *t*-test. The effect size of the significant differences was also analysed using Cohen's *d* (1992), whose parameters establish that an effect size below 0.20 is considered small, around 0.50 medium, and above 0.80 large. Secondly, Pearson's bivariate correlation analyses were carried out between the variables in the study. Thirdly, hierarchical regressions were carried out to analyse the predictive role of positive and negative affect, gambling motives and attachment in gambling, drugs, alcohol, video games and spending. For this purpose, each of the addictive behaviours was analysed using a model in which positive and negative affect was introduced in a first step, positive and negative affect and gambling motives in a second step, and positive and negative affect, gambling motives and attachment in a third step. For the second and third objectives, only the sub-sample of adolescents and young women with risk and problem gambling were considered. Due to the small size of that sample, the results were also replicated with the total sample to check the risk of Type 1 error (rejecting the null hypothesis when it is true).

## Results

Firstly, mean differences in attachment, gambling motives, positive and negative affect and the aforementioned addictive behaviours were analysed between possible problem gamblers and non-gamblers using Student’s *t-*test (Table 1). The results showed that female at- risk or with problematic gambling scored higher on gambling, drugs, compulsive spending, maternal attachment, and gambling motives (enhancement, social and coping motives). When analysing the effect sizes for the variables where significant differences were found, they were observed to be large for gambling, spending and enhancement motives, and medium for drugs, mother attachment, and social and coping motives.

**Table 1:** Mean differences between the group of problem gamblers and non-gamblers

	Problem gamblers	Non-gamblers	*t*(df)	*d*
	M(DT)	M(DT)		
1. Gambling severity	5.56(3.27)	.06(2.45)	− 10.51(1,38.054)*	1.90
2. Drugs	.90(1.16)	.44(.91)	− 2.12(1,34.690)*	.44
3. Spending	1.15(1.21)	.32(.67)	− 3.91(1,35.672)*	.84
4. Videogames	.73(1.18)	.60(1.12)	− .63(1,289)	.11
5. Alcohol	.77(1.17)	.41(.83)	− 1.64(1,32.566)	.35
6. Positive affect—2 weeks	27.88(6.95)	27.27(7.98)	− .43(1,311)	.08
7. Negative affect—2 weeks	20.16(6.47)	20.87(7.81)	.57(1,42.457)	.09
8. Positive affect—Now	25.09(7.97)	23.30(8.73)	− 1.12(1,306)	.21
9. Negative affect—now	18.13(8.42)	16.25(7.15)	− 1.34(1,291)	.24
10. Mother attachment	57.97(16.13)	64.15(14.01)	2.25(1,286)*	− .41
11. Father attachment	55.30(18.54)	58.07(16.33)	.87(1,288)	− .16
12. Peer attachment	65.39(14.18)	67.45(13.79)	.78(1,288)	− .15
13. Enhancement motives	8.54(3.72)	5.80(2.10)	− 4.27(36.915)*	.91
14. Social motives	6.34(1.80)	5.43(1.49)	− 2.74(1,36.60)*	.53
15. Coping motives	7.28(3.70)	5.36(1.36)	− 5.83(1,32.035)*	.69

*p < .05

Secondly, correlations between the study variables were analysed (Table 2). Gambling correlated positively with drugs, spending, negative affect (now), and enhancement and coping motives, and correlated negatively with maternal attachment. Drugs correlated positively with video games, alcohol, negative affect (now and past two weeks), and negatively with maternal and paternal attachment. Spending correlated positively with video games, alcohol, and enhancement and coping motives, and negatively with maternal and paternal attachment. Video games correlated positively with negative affect (last two weeks) and negatively with maternal attachment. Finally, alcohol abuse correlated positively with negative affect (last two weeks) and negatively with paternal attachment. The same results were obtained after replicating the results with the total sample, reducing the risk of Type I error (rejection of null hypothesis when it is true).

**Table 2:** Correlation among addictive behaviours, positive and negative affect, attachment and gambling motives in the subsample of female adolescents at-risk and with gambling problems

	1	2	3	4	5	6	7	8	9	10	11	12	13	14
1.Gambling severity	–													
2. Drugs	.16^**^	–												
3. Spending	.25^**^	.36^**^	–											
4. Videogames	.02	.15^**^	.21^**^	–										
5. Alcohol	.13^*^	.48^**^	.35^**^	.08	–									
6. Positive affect—Two weeks	.04	.04	.07	− .01	.10	–								
7. Negative affect—Two weeks	− .01	.23^**^	.11	.15^*^	.16^**^	.37^**^	–							
8. Positive affect—Now	.04	.02	.06	− .07	.09	.74^**^	.30^**^	–						
9. Negative affect—now	.12^*^	.20^**^	.14^*^	.11	.10	.28^**^	.70^**^	.34^**^	–					
10. Mother attachment	− .14^*^	− .19^**^	− .18^**^	− .17^**^	− .02	.23^**^	− .17^**^	.21^**^	− .08	–				
11. Father attachment	− .07	− .23^**^	− .13^*^	− .05	− .12^*^	.16^**^	− .22^**^	.17^**^	− .20^**^	.50^**^	–			
12. Peer attachment	− .04	.06	− .02	− .08	.11	.28^**^	.05	.24^**^	.04	.41^**^	.37^**^	–		
13. Enhancement motives	.27^**^	.04	.31^**^	.002	.10	.08	.02	.16^**^	.05	.07	.09	.02	–	
14. Social motives	.11	.02	.12	− .02	.03	.06	− .001	.07	.07	.10	.10	− .03	.71^**^	–
15. Coping motives	.37^**^	− .04	.32^**^	.04	.03	.03	− .005	.14^*^	.07	− .02	.04	− .04	.68^**^	.56^**^

^**^p < .01, *p < .05

Third, the predictive role of positive and negative affect, gambling motives and attachment in gambling, drugs, spending, alcohol, and video games was analysed using hierarchical regressions (Tables 3, 4, 5, 6). Hierarchical regression models were conducted, in which the first step included positive and negative affect; the second step included positive and negative affect and gambling motives; and the third step included positive and negative affect, gambling motives, and attachment. In the case of gambling, it was found fo be associated with social and coping motives. The first step explained a 3% of the variance, the second step explained a 34% of the variance, and the third step explained a 37% of the variance. The change in R^2^ was significant in the second step. In the case of drug abuse, it was associated with paternal attachment. The first step explained a 5% of the variance, the second step explained a 8% of the variance, and the third step explained a 16% of the variance. The change in R^2^ was significant in the third step. In the case of spending, it was associated with enhancement, social and coping motives. The first step explained a 1% of the variance, the second step explained a 22% of the variance, and the third step explained a 24% of the variance. The change in R^2^ was significant in the second step. In the case of alcohol, it was associated with enhancement motives, father attachment, and peer attachment. The first step explained a 3% of the variance, the second step explained a 7% of the variance, and the third step explained a 14% of the variance. The change in R^2^ was significant in the third step. In the case of video games, none of the models was significant. The same results were obtained after replicating the results with the total sample, reducing the risk of Type I error (rejection of null hypothesis when it is true).

**Table 3:** Hierarchical regressions of affect, gambling motives and attachment on gambling

	*t*	*Β*	SE B	*β*	*F*(df)	*R*	*R*^2^	adj.*R*^2^	Change in *R*^2^
*Step 3*					8.71(10,147)*	.61	.37	.33	.03
Positive affect—2 weeks	.96	.03	.03	.11					
Negative affect—2 weeks	− .68	− .02	.03	− .08					
Positive affect—now	− .69	− .02	.02	− .07					
Negative affect—now	.59	.02	.03	.06					
Enhancement motives	.80	.06	.07	.08					
Social motives	− 2.46*	− .26	.11	− .23					
Coping motives	6.78*	.53	.08	.60					
Mother attachment	− .78	− .01	.01	− .06					
Father attachment	− 1.72	− .02	.01	− .14					
Peer attachment	1.88	.02	.01	.14					

^*^p < .05

**Table 4:** Hierarchical regressions of affect, gambling motives and attachment on drugs

	*t*	*Β*	SE B	*β*	*F*(df)	*R*	*R*^2^	adj.*R*^2^	Change in *R*^2^
*Step 3*					2.70(10,143)*	.40	.16	.10	.08*
Positive affect—2 weeks	− .09	− .002	− .01						
Negative affect—2 weeks	.45	.01	.06						
Positive affect—now	1.07	.02	.14						
Negative affect—now	.28	.01	.04						
Enhancement motives	.81	.04	.10						
Social motives	.40	.03	.05						
Coping motives	− 1.79	− .09	− .18						
Mother attachment	.29	.002	.03						
Father attachment	− 3.36*	− .02	− .31						
Peer attachment	1.96	− 01	.17						

^*^p < .05

**Table 5:** Hierarchical regressions of affect, gambling motives and attachment on spending

	*t*	*Β*	*SE B*	*β*	*F(df)*	*R*	R^2^	adj.*R*^2^	Change in *R*^2^
*Step 3*					4.74(10,152)*	.49	.24	.18	.02
Positive affect – 2 weeks	− .57	− .01	.01	− .07					
Negative affect – 2 weeks	.63	.01	.01	.08					
Positive affect – now	.36	.004	.01	.04					
Negative affect—now	− .42	− .01	.01	− .05					
Enhancement motives	2.51*	.09	.04	.28					
Social motives	− 2.60*	− .13	.05	− .26					
Coping motives	3.63*	.12	.03	.35					
Mother attachment	.37	− .01	.01	− .08					
Father attachment	.17	− .01	.004	− .12					
Peer attachment	.34	.01	.01	.08					

^*^p < .05

**Table 6:** Hierarchical regressions of affect, gambling motives and attachment on alcohol

	*t*	*Β*	SE B	*Β*	*F*(df)	*R*	*R*^2^	adj.*R*^2^	Change in *R*^2^
*Step 3*					2.40(10,145)*	.38	.14	.08	.08*
Positive affect—2 weeks	.18	.003	.02	.03					
Negative affect—2 weeks	.38	.01	.02	.05					
Positive affect—now	.62	.01	.01	.08					
Negative affect—now	− .85	− .01	.02	− .11					
Enhancement motives	2.01*	.09	.04	.24					
Social motives	− 1.52	− .09	.06	− .17					
Coping motives	− .39	− .02	.04	− .04					
Mother attachment	1.28	.01	.01	.12					
Father attachment	− 2.98*	− .02	.01	− .28					
Peer attachment	2.21*	.01	.01	.20					

^*^p < .05

## Discussion

The first aim of this study was to analyse the differences between girls with risk and gambling problems, and the general female population without gambling problems, in addictive behaviours (gambling, video games, spending, alcohol and drugs), gambling motives (ENH, COP and SOC), attachment, and positive and negative affect. Adolescent females with gambling problems were found to score higher on gambling, drug use, compulsive spending, maternal attachment, and all gambling motives. To our knowledge, these data are novel as we found no studies that explore these variables in adolescent females specifically and conjointly. Nonetheless, the results are in line with previous studies conducted with young and adolescent populations, indicating that people with gambling problems obtain higher scores than the general population in gambling severity, gambling motives, attachment, drug abuse, and spending (Estévez et al., 2020a, b, c, d; Frisone et al., 2020; Jáuregui & Estévez, 2019).

Furthermore, the analyses in this study show that young women who met the criteria for risk or possible problem gambling presented significantly higher differences in gambling, spending and enhancement gambling motives, and slightly higher in the case of coping motives. Previous studies with samples of adult women have suggested that coping motives, that is, the tendency to gamble to cope with negative emotional states, are the main precursor of addiction in women (Fonseca et al, 2021; Lelonek-Kuleta, 2021); whereas enhancement motives, that is, the tendency to gamble to increase positive emotions, have been more closely associated with the severity of gambling behaviours in men (Stewart & Zack, 2008). However, both coping and mood enhancement motives have been conceptualised as motives for the regulation of affective states, and both of them have been associated with gambling disorder to a greater extent than did social motives (Cooper et al., 1995; Grande-Gosende et al., 2019). Ellenbogen et al. (2007), also indicated that the most striking result of their study was that traditional sex differences practically disappeared among adolescents with gambling problems, who showed similar gambling patterns. Although coping motives have been traditionally associated with gambling in women, this study also highlights the role of enhancement motives. There is evidence of an increase in impulsivity during adolescence, including positive urgency and sensation seeking, before stabilising in adulthood (Collado et al., 2014; Littlefield et al., 2016). However, this is still an under-explored area and requires further research.

Secondly, the relationship between addictive behaviours and the rest of the study variables was analysed in the group of girls with risk and possible gambling problems. We found that drug abuse, compulsive spending, and alcohol abuse correlated negatively with both maternal and paternal attachment, or paternal attachment; whereas gambling disorder and video games correlated negatively with maternal attachment. These results are consistent with previous studies showing that patterns of an insecure attachment style are related to the symptomatic expression of risky addictive behaviours in adolescents, as well as to increased susceptibility for the development of these behaviours in adulthood (Di Trani et al., 2017; Strathearn et al., 2019; Terrone et al., 2021). Similarly, several studies have indicated a close link between adult gamblers and subsequent pathological gambling in their children (Dowling, Merkouris, et al., 2017; Dowling, Shandley, et al., 2017).

Studies have shown mixed results regarding the influence of the specific attachment figure (i.e., maternal or paternal) in the development of different addictive behaviours (Estevez et al., 2017; Forrest & McHale, 2021). This study highlights the relationship between maternal attachment and gambling and video game disorders, which are increasingly similar in terms of their structural and addictive characteristics (Dowling et al., 2018; Griffiths & Wood, 2000). In this regard, a study conducted by Jáuregui and Estévez (2019) points out that maternal attachment predicts social and enhancement gambling motives, which could explain the positive relationship between gambling and enhancement motives, and the negative relationship between gambling and maternal attachment found in this study. However, the data are still limited and are based on samples composed predominantly of men. Therefore, a more consistent body of research that integrates gender perspective is needed to explore the specific role of attachment figures in addiction types, as well as its relationship with gambling motives.

To conclude, the third aim of the study was to examine the predictive role of positive and negative affect, gambling motives, and attachment in the aforementioned addictive behaviours. The results indicate that the predictive variables differ depending on the addiction type. In the case of substance addictions (i.e., alcohol and drugs), mainly the predictive role of attachment has been highlighted, whereas, in behavioural addictions (i.e., gambling and spending), gambling motives have been identified as the most important predictive variables. Although substance and behavioural addictions overlap in multiple neurobiological and clinical transdiagnostic features, these results reinforce previous studies suggesting that each addictive behaviour also has its own constellation of unique aetiological, personality, or clinical traits (Kim et al., 2020; Zilberman et al., 2018).

On the other hand, in the case of gambling, coping motives were shown to be the most important predictor, findings that are in line with previous literature on female gamblers (Fonseca et al, 2021; Stewart & Zack, 2008). By contrast, the analyses in this study do not show affect, neither positive nor negative, as a predictive variable of addictive behaviours in female adolescents. These results are novel and goes against previous literature because, to date, numerous studies have identified the gambling episode as an emotion regulation mechanism to alleviate and/or avoid an intense affect that the person is trying to avoid thinking about (Cicarelli et al., 2017; Hing et al., 2016). In view of the above, the relationship between affect and gambling is likely to be indirect and, therefore, probably mediated by gambling motives. In this sense, previous research has found a mediating relationship between affect and gambling severity through gambling motives, and have even hypothesised that the relationship between positive affect and gambling may be mediated by enhancement motives, while negative affect may be more closely related to coping motives (Ballabio et al., 2017; Kim et al., 2019; Takamatsu et al., 2016). Nonetheless, these findings may also suggest a differential profile of women suffering from problem gambling based on age (e.g. adults and youths), something that previous studies with other addictions have already demonstrated (Granero et al., 2014, 2018; Nicolai et al., 2012). However, more research is required to examine problem gambling from a gender- and developmental stage- perspective, to provide an effective social and healthcare response for this collective.

## Limitations

There are limitations that should be considered when interpreting its results. Firstly, the cross-sectional and mainly correlational design of the present study does not allow establishing interpretations regarding causality and the direction of the effects. In the future, longitudinal designs are needed to achieve an in-depth understanding and shed light on the interaction between the variables under scrutiny. Secondly, self-report measures were used, which could have biased the results. The inclusion of clinical and qualitative criteria to detect the presence or absence of addictive disorders could potentially complement the present data. Moreover, the sub-sample of problem gambling size is not large, and it is obtained from association centres and adolescent females from the general population who scored on the SOGS-RA as at-risk or potential problem gamblers. Therefore, this sample may present differential characteristics from other clinical samples (e.g., public hospitals, private therapy centres, people who are not yet in treatment, etc.). Nevertheless, the subclinical sample does allow us to explore risk factors that may precede the development of more severe addictive behaviours in the general population, which is beneficial for preventive purposes. Further research should probably focus on comparing different profiles of female problem gamblers, considering age, as well as socio-demographic and clinical characteristics, as the results of this study cannot be generalised to adult females. However, it should be pointed out that given the lack of research on adolescent females with gambling problems, adult females have been used on several occasions to compare the results of this study. We know that it is developmentally difficult to compare adolescents with adults, but we wanted to consider what we do know about other women with GD despite their age difference. Finally, the findings obtained in this study are based on sex differences, so it would be appropriate to carry out studies based on gender differences.

## Conclusion

Despite the limitations of this study, is there is a lack of studies exploring the profile of adolescents and young women with GD, as well as the comorbid presence of GD with substance-related problems and other behavioural addictions in this population. One of the main issues that emerges from these findings is the clear need to consider sex and age when designing treatments for people suffering from GD due to the clinical differences discussed throughout the manuscript. Furthermore, this study showed that the importance of the predictive variables for each addiction could differ from one addiction to another. These results are of great interest for prevention and intervention purposes. Given that higher co-morbidity in early ages is associated with worse prognosis and higher psychopathology in adulthood, the early detection, prevention and treatment of problems related to GD becomes essential.

## Recommendation

Many of the therapeutic interventions with adolescents with GD are based on group therapy. Therapy groups are a tool that have demonstrated numerous benefits, highlighting the therapeutic power of the shared experience. There are usually not enough young women to generate a group just with them, however, they do make mixed groups (i.e. with young women and men together) in which age and gambling is a common factor among them. In this sense, the group allow us to observe a micro-representation of how these young people develop their gender roles in society. This allows us to act directly on gender biases, enabling us to seek a balance between autonomy (something culturally associated with the male gender role) and care, interdependence and emotional expressiveness (culturally associated with the female gender role). It should also be noted that interventions with youth are especially preventive for their further development in adulthood. Finally, we make an invitation to think "with gender glasses", that is, to look beyond what is a mere biological sex difference, and to consider the educational and socialisation process that determines gender, as well as its relation to the development of the symptom.
